# Use of a Dermal Regeneration Template in a two-stage reconstruction after extensive skin surgery for Bowen's disease of the anterior neck - Case report^☆^

**DOI:** 10.1016/j.ijscr.2023.109208

**Published:** 2023-12-30

**Authors:** Davide Grassi, Marco De Monti, Vittoria Espeli, Laura De Pellegrin

**Affiliations:** a EOC – Mendrisio Beata Vergine Regional Hospital - Department of General Surgery, Switzerland; b EOC – Mendrisio Beata Vergine Regional Hospital - Oncology Institute of Southern Switzerland, Switzerland

**Keywords:** Bowen's disease, Dermal Regeneration Template, Skin cancer, Squamocellular carcinoma

## Abstract

**Introduction:**

Dermal Regeneration Templates may be used in the reconstruction of large skin defects after cutaneous malignancy excisions. Bowen's disease (BD; squamous cell carcinoma in situ) is a common and persistent condition that can be related to chronic sun damage, and consequently, is usually located on the head and neck area or on the lower limbs. Literature does not provide clear guidelines on the treatment of BD, limiting itself to describing a wide range of different methods that can be used, including surgery, laser therapy or topical options. However, large lesions tend to scar in the post-operative setting and hence are difficult to treat surgically.

**Presentation of case:**

In this paper the authors present a case of a male in his 60s, ASA III score, who presented with a history of histopathologically-confirmed squamocellular carcinoma in the neck and supraclavicular region. Due to recurrent carcinomas the patient was treated with an extensive skin excision and a successful reconstruction using a Dermal Regeneration Template. The work has been reported in line with the SCARE criteria.

**Discussion:**

The main surgical problem caused by BD is reaching complete oncological resection and, consequently, the need for extensive skin excisions.

**Conclusion:**

The use of the skin substitute resulted in a satisfactory functional and aesthetic result, with total clearance and no recurrence observed after 16 months.

## Highlights

• DRT may be used in the reconstruction of large skin defects after cutaneous malignancy excisions.
• DRT may be used in the therapy of Bowen’s disease
• DRT provides an efficient alternative reconstructive mechanism to extensive free-tissue transfer and offers a unique advantage in medically-complex patients.

## 1 Background

The skin is the prevalent site for primary malignant neoplasms and skin cancer has a higher incidence than all other forms of cancer combined [1,2]. Skin tumors are most frequent in photo-exposed areas, with the scalp being the prevalent location for soft tissue malignancies, and the number of cases increasing with age [3]. Large defects after resection according to oncologic criteria in the head and neck region historically implied the use of extended local flaps, skin grafts or healing by secondary intention [4]. Elderly patients, in particular, require a rapid procedure with an acceptable aesthetic and reliable functional outcome and for this reason the use of Dermal Regeneration Templates (DRT) for the reconstruction of full-thickness defects after skin cancer surgery have gained popularity [5]. In our case we used Integra®, which was initially approved by the FDA in 1996 for the treatment of major burn wounds, but since then its uses have been extended to the majority of extensive skin substance losses. Integra® is a bi-layered acellular matrix composed of an outer thin layer of semipermeable polysiloxane (silicone) that limits bacterial invasion and moisture absorption, and a thicker inner layer composed of porous type I collagen derived from bovine tendinous tissue, arranged in the form of a criss-cross network including glycosaminoglycans [6]. Its 3-D array structure mimics an extracellular matrix and a form of scaffold that enables various cell types, such as endothelial cells and fibroblasts, to migrate, resulting in the formation of a neodermis that, histologically and functionally, is very similar to autologous dermis. This form of DRT has a very low antigenicity and degrades in a controlled span of time of 3–4 weeks, being replaced by the host's collagen, without any added scar tissue [7]. After the removal of the silicone component, most cases require an epidermal autograft.

As stated above, sun exposure is one of the major risk factors that causes these soft tissue malignancies, including clinical features like Bowen's disease also known as “Dermatosis precancerosa” or “Cutaneous Cell Carcinoma In Situ (cSSC)” [8].

This malignant tumour arises from epidermal keratinocytes and presents heterogeneous forms of clinical manifestation, such as erythematous, well-demarcated, plaque or scaly patches. In addition, these lesions can be smooth, hyperkeratotic or ulcerated, and as they are usually asymptomatic, they are often not recognised in the early stages [9].

Although clinical and dermoscopic findings may strongly suggest a diagnosis of cSCC, a histopathologic examination is necessary in order to confirm the diagnosis. In fact, cSCC in situ (Bowen's disease) is diagnosed when a histopathologic examination reveals keratinocytic dysplasia involving the full thickness of the epidermis without infiltration of atypical cells into the dermis [10,11]. Keratinocytes are pleomorphic with hyperchromatic nuclei, and numerous mitoses are present. Frequently, there is an associated thickening of the epidermis (acanthosis), as well as hyperkeratosis and parakeratosis of the stratum corneum [10].

Histopathologic examinations are also useful for the assessment of perineural invasion, tumour differentiation, and tumour depth – important factors for tumour staging and prognosis [12]. We present a case of large Bowen's disease present for over 10 years in the anterior neck region, treated successfully using a Dermal Regeneration Template. To the best of our knowledge, the use of Integra® and a split-thickness skin graft in the management of this form of skin cancer surgery in the head and neck region has not been reported previously. The reported case has been managed in a public general hospital. This work has been reported in line with the SCARE criteria as well as the PROCESS Guidelines [12,13].

## 2 Case presentation

**Fig. 1:**
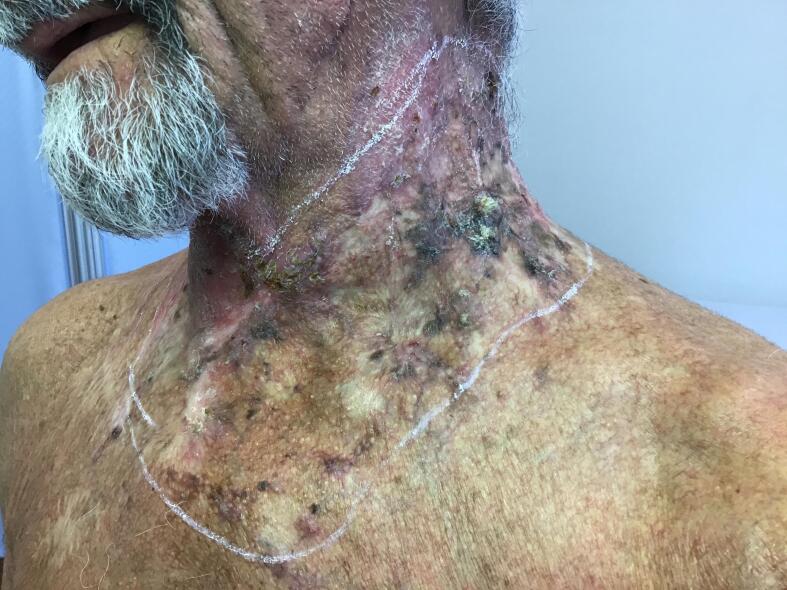
Extensive lesions in the anterior cervical region.

A male in his 60s, known for Chronic Obstructive Pneumopathy Disease (GOLD IIIb), previous rectal adenocarcinoma and peripheral arteriopathy treated with Aspirin, with an ASA III score, presented with a history of histopathologically-confirmed squamocellular carcinoma in the neck and supraclavicular region. In the last 10 years he had undergone local and extensive excisions of multiple lesions in the anterior neck region, which were treated locally with topical Imiquidmod, CO2 and laser therapy sessions, photodynamic therapy and cryotherapy. These therapeutical approaches, which were not carried out by our department, resulted in significant scar retraction, causing reduced neck extension. At the time the patient presented complete local remission, and so we decided on an autologous adipose tissue transplant, with the aim of regaining the full range of motion in this anatomical region. Despite a good functional outcome approximately 10 months after this surgical procedure, the patient presented multiple ulcers and neoplastic lesions at the surgical site (Fig. 1).

The work has been reported in line with the SCARE criteria.

## 3 Treatment

Due to the high rate of recurrence of this malignancy, we decided to perform a complete full-thickness skin resection of the area, according to oncological criteria.

**Fig. 2:**
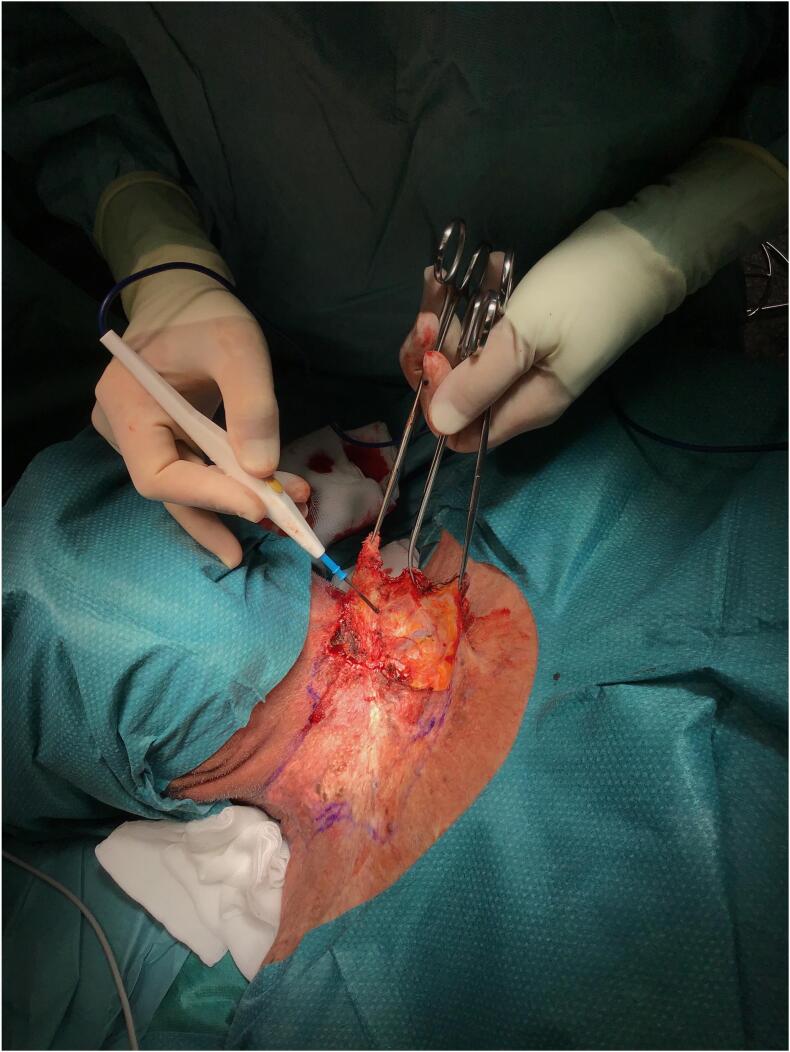
Surgical excision.

**Fig. 3:**
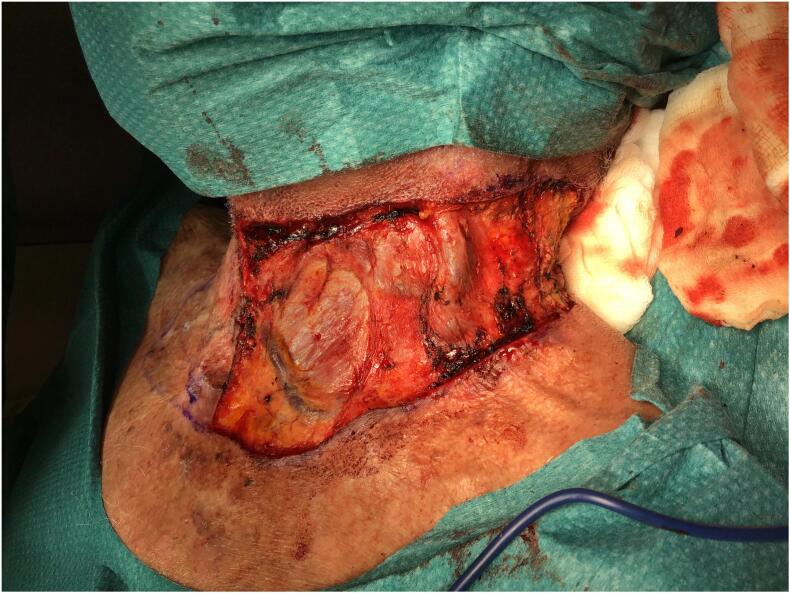
Resulting skin defect.

**Fig. 4:**
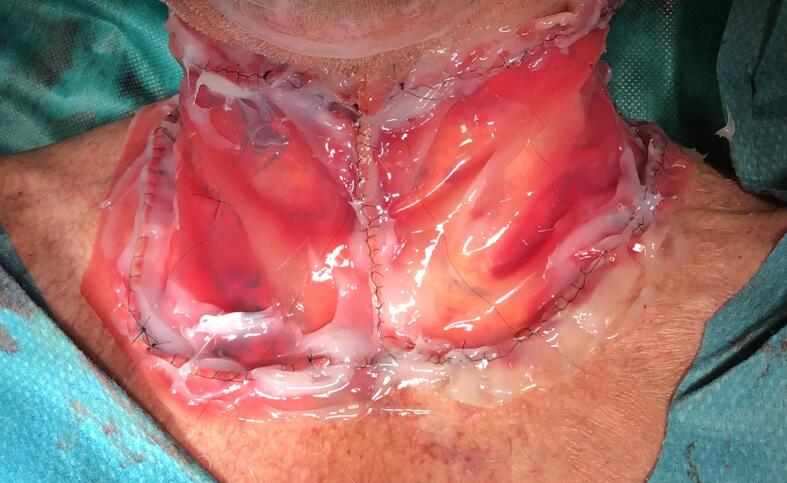
Implant of Dermal Regeneration Template (DRT).

**Fig. 5:**
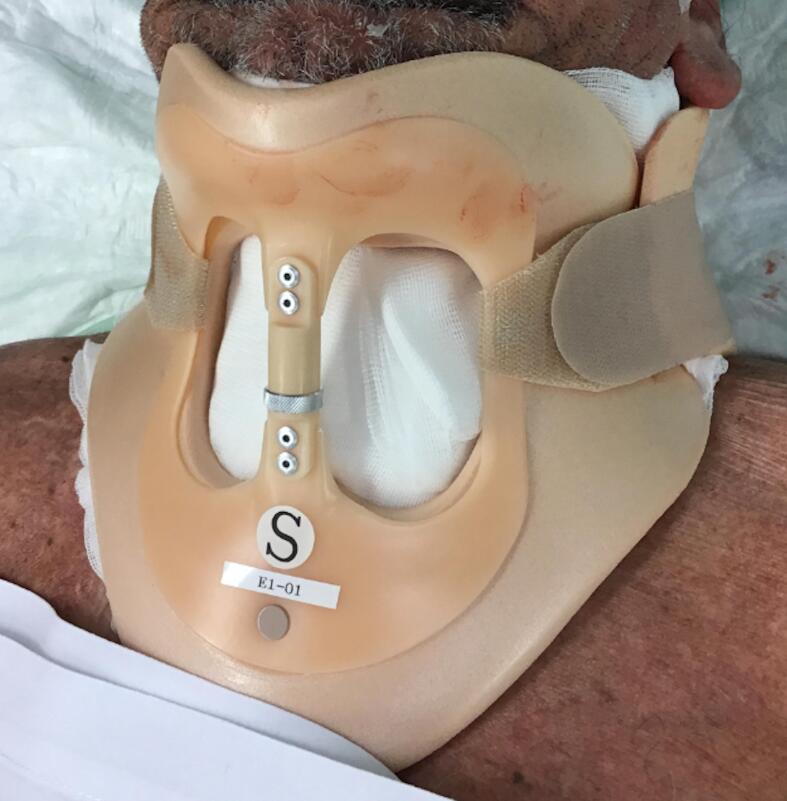
Compressive medication.

**Fig. 6:**
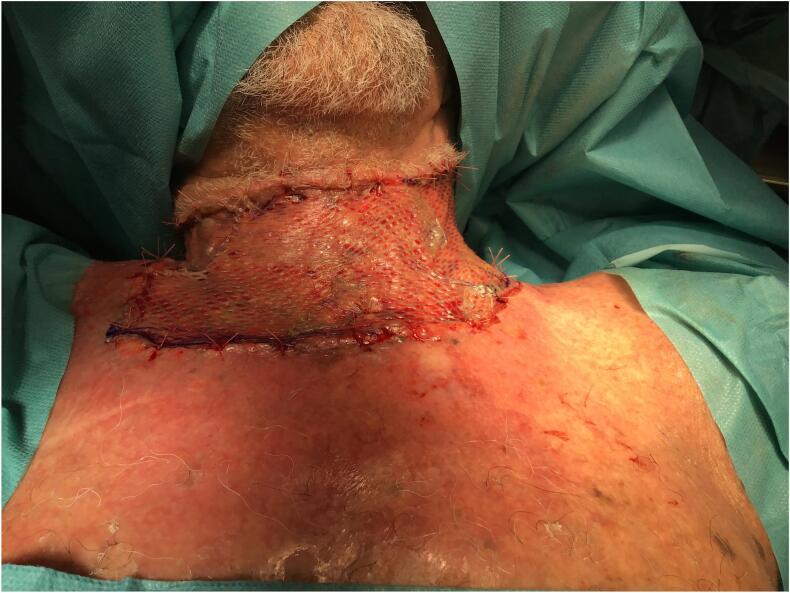
Split-thickness skin graft.

The skin defect extended vertically from the jugulum to the hyoid bone and bilaterally to the anterior border of the *M. trapezius*, which was then covered with Integra® Bilayer Matrix Wound Dressing (Integra LifeSciences Corp., Plainsboro, NJ, USA), a Dermal Regeneration Template (DRT), of 30x10cm. Our dermal matrix fixation protocol involved a continuous Ford-interlocking suture and a sealing dressing with fusidic acid unguent (Fig. 2, Fig. 3, Fig. 4). In order to achieve the effective compression of the operated area, with the consequent optimal adherence of the DRT on the wound bed, a Philadelphia neck collar was positioned (Fig. 5). The histopathological exam showed diffuse squamous cell carcinoma in situ, spreading bilaterally on the resected skin region, with free margins, and so we proceeded with the second reconstructive step three weeks after the application of the DRT, implanting a split-thickness skin graft, taken from the left lateral thigh (Fig. 6). After the split-thickness skin graft had completely healed, the patient quickly regained the full range of motion of his neck in ante- and retroversion, which was not possible in the pre-operative setting due to the skin contractions caused by the previous therapeutic approaches.

## 4 Outcome and follow-up

**Fig. 7:**
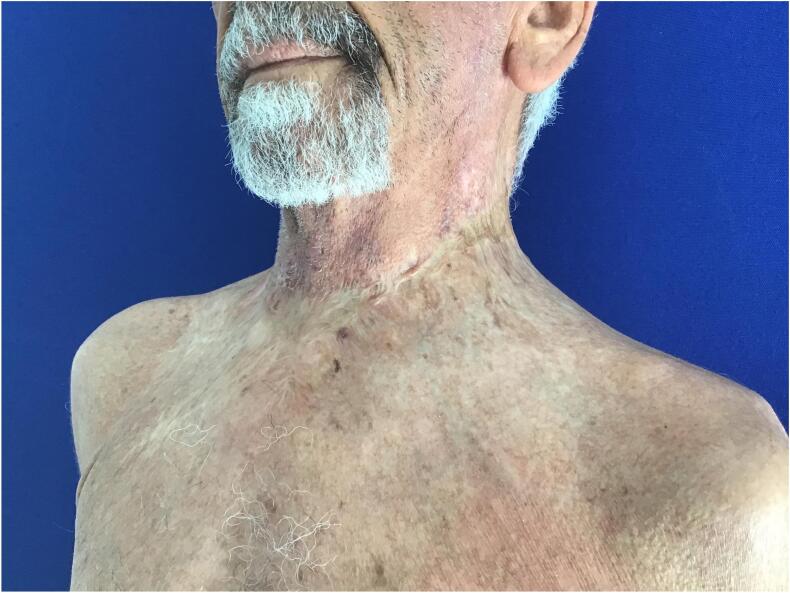
Final outcome after 16 month.

The patient was discharged from follow-up 16 months after the original treatment with a good aesthetic and functional outcome (Fig. 7).

## 5 Discussion

**Fig. 8:**
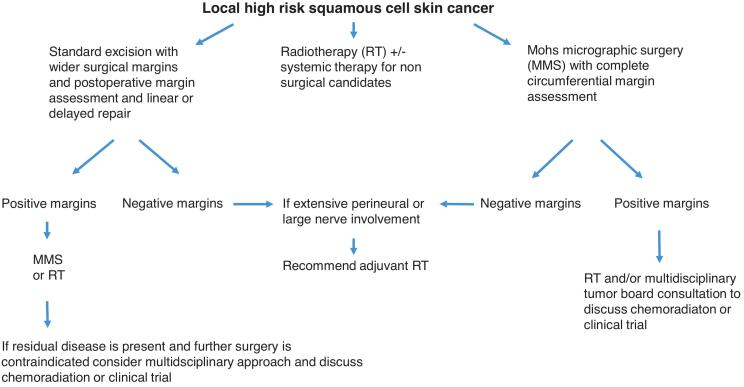
Therapeutic schema for local high risk squamous cell skin cancer (Created by D.Grassi MD).

Due to a lack of clear guidelines, the treatment of Bowen's disease includes a vast spectrum of options, including surgery, cryotherapy, radiation and photodynamic therapy, and as there are no strict indications for each technique, the choice of treatment often is at the discretion of the clinician (Fig. 8).

Bowen's disease, being a form of in situ SCC, meaning the whole epidermis is involved, can be eradicated completely by performing a full-thickness resection of the affected epidermal and dermal layer [8]. The reconstruction is performed autogenously by using a DRT and subsequently a split-thickness skin graft, taken from a healthy skin area. Further major advantages of this bi-layered template are its immediate availability, optimal scarring and the decreased requirement for donor tissue compared to major free-tissue transfer.

We are aware, of the downsides of this tow-staged surgical approach, which indeed implements the need for complex wound care, and goes along with an increased risk of infection and elevated costs. However due to the high recurrence rate and in this particular case multiple previous treatment attempts, this is justified, especially considering the long follow-up phase without recurrence as well as the aesthetic and functional outcome.

With regard to our clinical experience within the field of reconstructive surgery of the head and neck region and the current state of literature, the clinical cases in which the use of DRT is recommended are [9]:

- elderly or extremely elderly patients, who may present additional risk factors related to co-morbidity; (Table 1).

**Table 1:** Risk factors for local recurrence or metastases.

Criteria	Low risk	High risk
Anatomical localization	Area I < 20 mm Area II < 10 mm	Area I ≥ 20 mm Area II ≥ 10 mm Area III
Borders	Well-defined borders	Poorly defined
Neoplasm	Primary	Recurrent
Patient based		Immunosuppression Prior radiotherapy Rapid growing neoplasm Neurologic symptoms
Histology	Well or moderately differentiated <2 mm depth Clark level I,II,III	Poorly differentiated Acantholyc, adenosquamous, desmoplastic or metaplastic subtype ≥2 mm dept Clark level IV, V
- wide local excisions;
- failure of different therapeutic approaches;
- anatomical areas with a high mobility requirement.

## 6 Conclusion

To summarize, even after a diagnosis has been established, choosing the right treatment may be challenging due to poor clinical evidence. There is also a lack of comparison between treatments, as most studies only provide data on the rate of remission, not the functional final outcome.

Hence, further research is required in order to establish the appropriate approach to be taken when treating Bowen's disease.

To conclude, we consider that the acellular dermal matrix provides an efficient alternative reconstructive mechanism to extensive free-tissue transfer and offers a unique advantage in medically-complex patients.

## Consent

Written informed consent was obtained from the patient for publication of this case report and accompanying images. A copy of the written consent is available for review by the Editor-in-Chief of this journal on request.

## Ethical approval

Case report does not need ethical approval.

However, this is a case report and is not a retrospective or even prospective study. It does not involve any experimentation or double-blind treatments.

The treatments carried out were agreed with the Patient and fully explained, we obtained broad consent for the surgical procedures and we obtained total consent from the Patient himself for the publication of the case and for the dissemination of the images. In such cases these are not topics to be submitted to the ethics committee.

## Funding

No founding.

## CRediT authorship contribution statement

Davide Grassi Study concept or design.

Marco De Monti Plastic Surgeon.

Vittoria Espeli Data analysis or interpretation.

Laura De Pellegrin Writing the paper.

## Guarantor

Davide Grassi.

Marco De Monti.

## Research registration number

It isn't a perspective study, but a single case report.

## Declaration of competing interest

All authors disclose any financial and personal relationships with other people or organisations that could inappropriately influence their work.
